# Standing of nucleic acid testing strategies in veterinary diagnosis laboratories to uncover *Mycobacterium tuberculosis* complex members

**DOI:** 10.3389/fmolb.2014.00016

**Published:** 2014-10-15

**Authors:** Pedro Costa, Ana Botelho, Isabel Couto, Miguel Viveiros, João Inácio

**Affiliations:** ^1^Instituto Nacional de Investigação Agrária e Veterinária IPLisboa, Portugal; ^2^Grupo de Micobactérias, Unidade de Microbiologia Médica, Instituto de Higiene e Medicina Tropical da Universidade Nova de LisboaLisboa, Portugal; ^3^Centro de Recursos Microbiológicos (CREM), Universidade Nova de LisboaCaparica, Portugal; ^4^Centro de Malária e Outras Doenças Tropicais, Instituto de Higiene e Medicina Tropical da Universidade Nova de LisboaLisboa, Portugal; ^5^School of Pharmacy and Biomolecular Sciences, University of BrightonBrighton, UK

**Keywords:** nucleic acid testing, molecular diagnostics, *Mycobacterium tuberculosis* complex, *Mycobacterium bovis*, tuberculosis, bovine tuberculosis, SWOT analysis

## Abstract

Nucleic acid testing (NAT) designate any molecular approach used for the detection, identification, and characterization of pathogenic microorganisms, enabling the rapid, specific, and sensitive diagnostic of infectious diseases, such as tuberculosis. These assays have been widely used since the 90s of the last century in human clinical laboratories and, subsequently, also in veterinary diagnostics. Most NAT strategies are based in the polymerase chain reaction (PCR) and its several enhancements and variations. From the conventional PCR, real-time PCR and its combinations, isothermal DNA amplification, to the nanotechnologies, here we review how the NAT assays have been applied to decipher if and which member of the *Mycobacterium tuberculosis* complex is present in a clinical sample. Recent advances in DNA sequencing also brought new challenges and have made possible to generate rapidly and at a low cost, large amounts of sequence data. This revolution with the high-throughput sequencing (HTS) technologies makes whole genome sequencing (WGS) and metagenomics the trendiest NAT strategies, today. The ranking of NAT techniques in the field of clinical diagnostics is rising, and we provide a SWOT (Strengths, Weaknesses, Opportunities, and Threats) analysis with our view of the use of molecular diagnostics for detecting tuberculosis in veterinary laboratories, notwithstanding the gold standard being still the classical culture of the agent. The complementary use of both classical and molecular diagnostics approaches is recommended to speed the diagnostic, enabling a fast decision by competent authorities and rapid tackling of the disease.

## Tuberculosis and the *Mycobacterium tuberculosis* complex

Tuberculosis is an epidemic and serious infectious disease of global proportions, responsible for the death of one to two million people per year (WHO, 2011). The disease also affects wild and domestic animals, particularly livestock. Bovine tuberculosis is a zoonosis with high socio-economic impacts due to the low productivity of affected cattle, to the imposed restrictions on animal trade and products thereof, and due to the costs associated with the implementation of control and eradication programs (Schiller et al., 2011). This disease also raises important public health concerns, particularly in developing countries, where the main routes of transmission to humans are the contact with infected animals and ingestion of unpasteurized dairy products (Kubica et al., 2003; Etter et al., 2006; Rua-Domenech, 2006; Rodríguez et al., 2009; Michel et al., 2010; Pérez-Lago et al., 2013; Torres-Gonzalez et al., 2013). Nevertheless, there is some evidence of possible person-to-person transmission of the disease (Evans et al., 2007; Sunder et al., 2009). In most developed countries, bovine tuberculosis has been tackled during the last decades by costly eradication programs. However, the eradication of the disease has been hampered in many countries by the presence of wild animals which act as reservoirs of disease, among which are the European badger (*Meles meles*), the possum (*Trichosurus vulpecula*), the bison (*Bison bison*), the African buffalo (*Syncerus caffer*), the white-tailed deer (*Odocoileus virginianus*) and the wild boar (*Sus scrofa*) (Santos et al., 2010; Maas et al., 2013; Hardstaff et al., 2014).

Tuberculosis is caused by members of the *Mycobacterium tuberculosis* complex (MTC), a group of closely-related species including: *M. tuberculosis* (the predominant cause of human tuberculosis); “*M. canettii*” (a very rare MTC biotype); *M. africanum* (mainly associated to human tuberculosis in Africa); *M. pinnipedii* (the cause of endemic tuberculosis in several seal species); *M. microti* (with bank voles and other small rodents as natural hosts, rarely identified from other mammals); *M. mungi* (associated to tuberculosis in banded mongooses in Botswana); *M. orygis* (a recently described and less known rare species, with oryxes, waterbucks, and gazelles as potential hosts in Africa, and bovines and rhesus monkeys in South Asia); and *M. bovis* and *M. caprae* (the worldwide predominant cause of bovine and goat tuberculosis, respectively, but also causing disease in a wide range of domestic and wild animals, including humans) (Huard et al., 2006; Smith et al., 2009; Alexander et al., 2010; van Ingen et al., 2012; Broughan et al., 2013; Rodriguez-Campos et al., 2014). Although a rare event, infection with *M. tuberculosis* may occur in animals living in close contact with humans, such as pets, pigs, cattle and captive animals (Erwin et al., 2004; Amado et al., 2006; Schmidt et al., 2008; Mohamed et al., 2009; Botelho et al., 2014; Rodriguez-Campos et al., 2014). Although the differences in their epidemiology, namely geographic distribution and host preferences, virulence traits and antimicrobial susceptibility patterns (Brosch et al., 2000), MTC species share more than 99.9% genomic sequence homology, with very low levels of genetic diversity at the nucleotide level, and present identical 16S ribosomal gene sequences (Sreevatsan et al., 1997; Mostowy et al., 2005). The genome of these species has a size of approximately 4.4 million base pairs, which reflects the complexity of their life cycles as facultative intracellular parasites, and contains a higher GC content of about 65%. The exchange of genetic material by horizontal gene transfer (HGT) events is apparently very rare among these species, which results in their mainly clonal evolution (Coros et al., 2008). However, recent whole-genome based studies have suggested that HGT may not be as rare as previously thought in MTC (Veyrier et al., 2009; Supply et al., 2013; Wang and Behr, 2014). The ancestor of all tubercle bacilli probably underwent major events of genetic exchange with unrelated environmental bacteria, which might have contributed to the evolution of those species (Gutierrez et al., 2005; Becq et al., 2007; Jang et al., 2008; Veyrier et al., 2011; Namouchi et al., 2012). Remarkable, some genomic islands were found to contain genes with putative or documented virulence functions, notably the Rv0986–8 operon, probably acquired from a gamma-proteobacterial species and involved in the host cell parasitism (Rosas-Magallanes et al., 2006; Becq et al., 2007; Jang et al., 2008). The lack of more abundant HGT events among the MTC members may be a consequence of the organisms' solitary lifestyles within their hosts, preventing their contact with other bacterial species (Gutierrez et al., 2005; Smith et al., 2006; Jang et al., 2008).

The designation of the different members of the MTC as distinct formal species is somewhat controversial and challenges the usual concept of bacterial species, given the very reduced genome sequence diversity and the absence of major phenotypic differences within the complex. Indeed, these mycobacteria may be differentiated in species, subspecies or even host-adapted ecotypes depending on the strict taxonomical criteria used for interpretation (Rodriguez-Campos et al., 2014). Nevertheless, although genetically very similar, MTC members can be distinguished by stable molecular differences, such as single nucleotide (SNPs) and large sequence (LSPs) polymorphisms, whose analysis have been the basis of several evolutionary studies about these mycobacteria (Brosch et al., 2002; Mostowy et al., 2002, 2005). Comparative genomic analysis evidenced that modern MTC species probably evolved from a common ancestor through the accumulation of sequential and irreversible genomic deletions, named Regions of Difference (RDs), which has been important in generating genetic diversity within these closely related mycobacteria (Mahairas et al., 1996; Brosch et al., 1998, 2002; Behr et al., 1999; Gordon et al., 1999; Mostowy et al., 2002). The pattern of the presence or absence of these RDs in the genome of MTC members provides a molecular signature that can discriminate among these mycobacteria (Brosch et al., 2002; Mostowy et al., 2002; Huard et al., 2006; van Ingen et al., 2012). Other molecular markers, such as deletions of spacer sequences of the Direct Repeat (DR) locus and specific SNPs corroborate this discrimination among MTC members (Gutacker et al., 2002; Baker et al., 2004; Gutierrez et al., 2005; Huard et al., 2006; Smith et al., 2006; Alland et al., 2007). MTC members harboring animals as their main hosts share the absence of RD9 (present in *M. tuberculosis*) and other common features such as the deletion of specific DR spacers and common SNPs (Smith et al., 2006, 2009).

## Conventional approaches for the confirmation of tuberculosis in animals

In cattle, symptoms of tuberculosis often manifest later, so the clinical diagnosis is rare, especially in the course of control and eradication plans. In most developed countries, eradication plans of bovine tuberculosis mostly involve the culling of reactor animals and the laboratory testing of suspect tissue samples for the definitive confirmation of the presence of MTC, particularly of *M. bovis*. The screening of tuberculosis in live animals depends on immunological assays such as the single intradermal comparative cervical tuberculin (SICCT) and the interferon-gamma (IFN-γ) tests, which are based on the detection of cell-mediated immune responses. SICCT test is based in an observed delayed hypersensitivity reaction after an intradermal injection of bovine and avian tuberculin in animals. When tuberculin is injected into an animal whose immune system has been sensitized by infection with *M. bovis*, or by exposure to cross-reacting antigens, it triggers an inflammatory response and swelling at the injection site (Rua-Domenech et al., 2006). However, the sensitivity of this test is moderate with estimates typically in the range 50–60%. The IFN-γ test is an enzyme immunoassay based on the evaluation of cytokine released by lymphocytes previously sensitized with tuberculin and is generally used as a complement of the SICCT test (Gormley et al., 2006). The IFN-γ test is generally accepted to have a higher sensitivity than the SICCT test but does not have a sufficiently high specificity to allow its use as a general screening tool. Antibodies in measurable quantities are only produced in later stages of the disease (Neill et al., 2001; Pollock and Neill, 2002) and, consequently, diagnostic methods based on the humoral response usually yield low sensitivities (Pollock et al., 2005). Nevertheless, these tests may be of value in identifying animals with more advanced disease and therefore more likely to be infectious.

The detection of MTC bacteria in animal tissues is mainly based in lengthy and cumbersome conventional methods, involving the examination of Ziehl-Neelsen stained smears, histopathology, and culture in selective media, followed by biochemical or molecular identification of typical mycobacteria colonies. The microscopic observation of acid-fast mycobacteria is non-specific and highly insensitive, particularly in the case of paucibacillary forms of tuberculosis. Culture remains the gold-standard method to confirm tuberculosis infection but requires several weeks to obtain positive results due to the extremely fastidious growth of tuberculous mycobacteria. A variety of different media has been developed for the cultivation of mycobacteria including egg-based media (e.g., Stonebrink's medium and Löwenstein–Jensen with added pyruvate), agar-based media eventually enriched with serum or blood (e.g., Middlebrook 7H10 and 7H11), and liquid media (e.g., Middlebrook 7H9) (Gormley et al., 2014). The introduction of automatic radiometric (e.g., BACTEC 460) and fluorometric (e.g., BACTEC 9000MB or MGIT 960) equipments contributed to shorten the culture time (Drobniewski et al., 2003; Hines et al., 2006). Culture has a high specificity but is not particularly sensitive, especially when using lymph node samples without observable lesions, even when animals are positive for SICCT or IFN-γ tests (Pollock and Neill, 2002; Rua-Domenech et al., 2006). The results of culture assays may be also affected by several factors, such as the decontamination procedures of samples, which can also have a harmful effect on mycobacteria, as well as the growth media and incubation conditions used and the constrained distribution of mycobacteria in tissues (Corner, 1994; Corner et al., 2012; Gormley et al., 2014). Moreover, conventional laboratory diagnosis does not routinely discriminate between the species of the MTC. In most circumstances, phenotypic and biochemical assays able to discriminate MTC members, such as the assessment of oxygen requirements, niacin accumulation, nitrate reductase activity, resistance to pyrazinamide or use of glycerol and pyruvate by the MTC strains (Wayne and Kubica, 1986; Grange et al., 1996) are slow, may yield ambiguous results and may be limited by a degree of technical complexity that rule out its implementation in routine diagnostics (Niemann et al., 2000).

## Nucleic acid testing of veterinary-relevant MTC bacteria

The field of molecular diagnostics met tremendous technological developments in the last two decades, and several strategies and applications have been described to identify and characterize MTC mycobacteria cultures and to directly detect these organisms from a wide variety of matrices, including animal tissues. The first molecular diagnostic methods have emerged in the 70s of the last century, when DNA probes labeled with radioactive isotopes were used to detect complementary DNA. These methods have evolved after the introduction of restriction enzymes, used to cleave the microbial genomes. The resulting fragments were then separated by gel electrophoresis, transferred to a membrane and subjected to hybridization with labeled DNA probes in a procedure known as Southern blotting in tribute to the original inventor of the technique (Southern, 1975). Over time, the hybridization procedures were improved, including the labeling of DNA probes with reporter molecules safer and easier to use, such as biotin or digoxigenin (Langer et al., 1981), allowing a colorimetric detection of hybridization results, or with fluorescein and other luminescent molecules (chemiluminescent reaction). Several applications are described in the literature where the direct hybridization with DNA probes allowed the identification of cultured *M. bovis* and other MTC members (Picken et al., 1988; Thierry et al., 1992; Cousins et al., 1993; Fisanotti et al., 1997). However, despite the high specificity, the applications based on direct hybridization were never really adopted in the routine clinical diagnosis of animal tuberculosis, since they are very labor intensive, costly, slow and insensitive.

The introduction of *in vitro* nucleic acids amplification technologies enabled the development of new tools for the rapid, sensitive, and specific detection and identification of pathogenic microorganisms, including tuberculous mycobacteria. The most commonly used technology is based on the polymerase chain reaction (PCR), introduced in the mid-80s of last century (Saiki et al., 1985, 1988). The first applications of PCR for the detection and identification of veterinary-relevant MTC members were published soon after (Cousins et al., 1991; Plikaytis et al., 1991; Wards et al., 1995). The real-time PCR was introduced in the mid-90s of last century (Heid et al., 1996; Williams, 2009) and was a very significant advancement in PCR technology that greatly contributed to a wider use of molecular diagnostics technologies. Real-time PCR also allows quantifying the numbers of copies of the template DNA initially present in a sample. Although the significant advances in the development of novel molecular diagnostic assays toward a faster and accurate detection of MTC in human samples, only a few assays have been described for detecting these agents directly in animal tissues, particularly in fresh tissues from livestock (Coetsier et al., 2000; Roring et al., 2000; Taylor et al., 2001, 2007; Parra et al., 2008; Costa et al., 2013a; Araújo et al., 2014). Most of these molecular approaches are PCR-based and target specific polymorphisms, insertion sequences, particularly the IS*6110*, and RDs in the genome of MTC members (Liébana et al., 1995; Wards et al., 1995; Miller et al., 1997; Niemann et al., 2000; Taylor et al., 2001, 2007; Pounder et al., 2010; Thacker et al., 2011; Reddington et al., 2012). A higher number of PCR-based applications were described for the identification of MTC members using DNA extracted from cultures as template. Approaches such as the analysis of SNPs of the *gyrB* gene, and of other genes, or the assessment of the structure of variable DRs or of LSPs in the genome of MTC members have been described that effectively discriminate between these species (Niemann et al., 2000; Parsons et al., 2002; Huard et al., 2003; Djelouadji et al., 2008; Pinsky and Banaei, 2008; Halse et al., 2011). Several RDs-targeted assays have been proposed for MTC species discrimination, usually involving standard PCR reactions and analysis of products by gel electrophoresis (Warren et al., 2006; Chen et al., 2007; Allix-Béguec et al., 2010), real-time PCR using intercalating fluorescent dyes and melting curve analysis (Pinsky and Banaei, 2008; Pounder et al., 2010) and, to a lesser extent, dual labeled hydrolysis probes (Halse et al., 2011; Reddington et al., 2011, 2012; Costa et al., 2014a,b).

In the context of molecular microbiological diagnostics, the DNA fragments amplified by PCR, or by any other nucleic acid amplification technology, can also be analyzed by hybridization with DNA probes immobilized on solid supports (reverse hybridization) such as nylon or nitrocellulose membranes. The reverse hybridization technology enables the detection of multiple genomic targets in a single test, a format commonly referred to as macroarray in which different probes are immobilized in specific positions of membranes as dots (dot blots) or lines (line blots). The target DNAs are labeled during its amplification, for example using biotinylated primers, and the occurrence of hybridization with their complementary probes is signalized by an enzymatic step yielding a colorimetric or chemiluminescent signal. Several systems based on this format have been described for the detection, identification and even typing (e.g., spoligotyping) of veterinary-relevant MTC members and commercial diagnostic systems are also available (Padilla et al., 2003, 2004; Duarte et al., 2008).

## Major challenges of tuberculosis nucleic acid testing

It is widely recognized that the analytical sensitivity of amplification-based nucleic acid testing is extremely high, often allowing the detection of single copies of the target nucleic acids in the reaction mixture. However, the performance of these methods is many times unsatisfactory when used for the direct detection of pathogen's nucleic acids directly in samples, yielding false negative results. A problem still largely unsolved concerns the processes of extraction and purification of nucleic acids from biological samples, particularly from tissues. Indeed, most of the amplification-based assays described for detecting MTC nucleic acids directly in fresh or formalin-fixed paraffin-embedded animal tissues only yield a moderate sensitivity, usually up to 75% when compared to the reference bacteriological culture, particularly when testing tissues without the characteristic lesions (Liébana et al., 1995; Coetsier et al., 2000; Roring et al., 2000; Taylor et al., 2001, 2007; Parra et al., 2008; Thacker et al., 2011). This limitation is mostly related to the increased complexity for disrupting and recovering genomic DNA from the tough mycobacterial cells, which harbor complex walls, and to the inefficiency of the extraction procedures from affected animal tissues, especially those exhibiting strong fibrosis, calcification, and with no histological evidence of acid-fast bacteria (Liébana et al., 1995; Taylor et al., 2007; Amaro et al., 2008; Parra et al., 2008; Costa et al., 2013a; Radomski et al., 2013). The presence of amplification inhibitors in crude tissue extracts, and of large amounts of co-extracted host eukaryotic DNA, often represent an additional problem. Target mycobacteria are located within phagosomes, within a host cell and within a granuloma, making DNA extraction from these organisms more challenging (Radomski et al., 2013). The availability of efficient, simple to use and affordable mycobacterial nucleic acids extraction processes adapted to difficult biological matrices, such as animal tissues, is still an unmet need that has hindered a more widespread routine use of molecular methods for the detection of MTC members in the veterinary microbiology laboratory (Cunha and Inácio, 2014).

Important in the context of developing nucleic acid testing assays for tuberculosis is also the availability of adequate gold standard diagnostics methods to validate and assess the performance of those assays (Cunha and Inácio, 2014). The culture of tissue samples for the isolation of MTC members, followed by molecular or biochemical identification procedures, is usually the gold-standard method to validate alternative diagnostic assays. However, it is widely known that bacteriological culture is slow and laborious and can yield ambiguous or false-negative results, e.g., due to the presence of non-viable mycobacteria, raising concerns about its effectiveness as comparison reference method (Liébana et al., 1995; Santos et al., 2010; Costa et al., 2013a). As mentioned above, the results of culture assays may be affected by several factors, including the constrained distribution of mycobacteria in affected tissues (Corner et al., 2012; Gormley et al., 2014).

## Re-emerging strategies to uncover animal-relevant MTC members

An option that has been explored to enhance the sensitivity of PCR techniques for detecting MTC bacteria directly from biological samples involves the implementation of more effective mycobacterial DNA extraction and purification methods. For example, Taylor and colleagues were able to increase the *M. bovis* detection sensitivity by PCR in bovine tissues, with visible lesions, from 70 to 91%, after the inclusion of an additional cell disruptive step of freeze-thaw cycles with liquid nitrogen in the DNA extraction process (Taylor et al., 2007). Mechanical disruption using bead-beating approaches was also shown to be more effective for the recovery of mycobacterial DNA from tissues (Amaro et al., 2008; Radomski et al., 2013). The use of sequence capture or immunomagnetic separation (IMS) approaches was also previously proposed for recovering higher yields of mycobacterial DNA, particularly of *M. bovis*, from samples such as tissues, soil and feces (Roring et al., 2000; Taylor et al., 2001; Sweeney et al., 2006, 2007; Parra et al., 2008; Garbaccio and Cataldi, 2010), with different degrees of success. Noteworthy, IMS strategies specifically targeting *M. bovis* were very recently described by Stewart et al. (2013), allowing great sensitivity improvements for the culture and molecular detection of this mycobacterium from tissue specimens. IMS is a physical cell separation technique able to selectively capture and concentrate MTC bacteria from complex matrices using magnetic beads coated with specific polyclonal or monoclonal antibodies or other binders (Stewart et al., 2013; Grant and Stewart, 2014). After the nucleic acid extraction from the concentrated whole cells, captured on the magnetic beads, genomic targets such as the MTC-specific IS*6110* can be analyzed by PCR or other molecular techniques (Stewart et al., 2013; Grant and Stewart, 2014). Nevertheless, albeit promising, these DNA extraction approaches involve additional experimental steps and complexity, and usually require more expensive equipments and consumables, which currently limit their wider use for the routine laboratorial diagnosis of animal tuberculosis.

An established PCR-based strategy to increase the detection sensitivity thresholds of low-copy number DNA targets is the nested PCR, although this higher sensitivity needs to be balanced against the associated increased risk of cross contamination of samples. Thus, the use of nested PCR approaches, as for any diagnostics techniques, requires very strict good practice standard conditions for molecular analysis and an effective quality assurance and control (Costa et al., 2013a, 2014b; Kozlovac and Schmitt, 2014; Saunders and Sharp, 2014). Previous studies found no significant improvements in the detection of MTC members in animal tissue samples when using nested PCR assays, including with real-time PCR formats (Wards et al., 1995; Taylor et al., 2007; Thacker et al., 2011). However, it is widely know that the performance of PCR detection systems is highly dependent on the efficiency of the primers and probes, even when using the same genomic targets such as the MTC-specific IS*6110* element (Savelkoul et al., 2006). Recently published studies, including from our group, highlighted the usefulness of re-emerging nested PCR strategies, including a real time PCR format, for a more sensitive detection of MTC genomic targets directly in animal tissue extracts (Costa et al., 2013a, 2014b; Araújo et al., 2014). Costa and colleagues optimized an IS*6110*-targeted nested real time PCR approach allowing the direct detection of MTC members in animal tissue specimens, particularly from bovine, with very high sensitivity, specificity and positive and negative predictive values (Costa et al., 2013a). The inclusion of a first step of conventional IS*6110*-targeted PCR amplification, whose product is then used as template for a second *Taqman*®-based real time PCR, allowed to increase the sensitivity of the detection assay to near 100% (using culture as gold-standard). The duplex use of a β-actin gene targeted probe, with complementary targets in most mammals, allowed the assessment of amplification inhibitors in the tissue samples. Other recently published study by Araújo and colleagues described a similar nested real time PCR approach targeting the *TbD1* region of *M. bovis* in bovine and bubaline tissue homogenates (Araújo et al., 2014). The *TbD1* region appears to be present exclusively in the genomes of *M. bovis, M. africanum*, “*M. canettii*” and in ancestral strains of *M. tuberculosis* (Brosch et al., 2002), the last three taxa being very infrequently or not associated at all with infection in animals.

The discrimination between MTC members is important for an accurate diagnosis and epidemiological assessment of mycobacterial disease. In the last two decades, the accumulating knowledge of the nucleotide sequences of several genes, and of the whole genomes, of these species has allowed the development of novel molecular assays for their identification. As mentioned earlier in this review, the pattern of the presence or absence of specific RDs in the genome of MTC members provides a highly specific molecular signature that can accurately discriminate among these species. Furthermore, targeting LSPs instead of SNPs (e.g., in *gyrB* and other genes) to discriminate between MTC members may allow the development of more robust PCR-based assays for use in the routine diagnostics framework of veterinary laboratories. PCR-based detection assays targeting SNPs are more difficult to optimize and may be less robust, requiring a much more strict control of the experimental conditions to maintain the specificity. Several strategies targeting specific RDs were recently described for the identification of MTC members, also by our group, that focused mainly in the species of the complex most commonly associated with tuberculosis in livestock and other animals (Costa et al., 2014a,b). A two-step identification strategy was thus developed, comprising two sequential *TaqMan*-based multiplex real-time PCR reactions. The first step allows the identification as MTC, by targeting the IS*6110* element, or as a mycobacterial species, if only a 16S rDNA product is detected in the duplex amplification reaction. If a MTC member is identified, the subsequent RDs-targeted triplex real time PCR allow the identification to the species level as *M. bovis, M. bovis* BCG, *M. caprae* or *M. tuberculosis*, according to their distinct patterns of presence or absence of RD1, RD4 and RD9 loci. This assay is faster and simpler than other SNPs-targeted restriction analysis or reverse line probe hybridization-based molecular assays, since it bypasses any post-amplification experimental steps.

## Future prospects for the MTC nucleic acid testing

Nucleic acid testing assays for pathogens detection and identification are still mostly “in house” optimized in veterinary diagnosis laboratories, usually demanding highly qualified personnel and sophisticated and expensive facilities, equipment and consumables (Cunha and Inácio, 2014). Therefore, it is necessary that these technologies become simpler, standardized, and affordable to make them effectively and widely used in routine clinical laboratories, including in low-resource laboratories and in the developing world where animal and human tuberculosis is still a huge scourge. Isothermal nucleic acids amplification processes, such as Loop-Mediated Isothermal Amplification (LAMP) (Notomi et al., 2000), could facilitate the integration of nucleic acid testing strategies into bench molecular diagnostics kits independent of the utilization of sophisticated equipments (Niemz et al., 2011). The high potential of LAMP for the development of improved nucleic acid detection strategies fully justifies the increasing number of reports on its utilization, including for the detection of *M. tuberculosis* in sputum (Iwamoto et al., 2003; Boehme et al., 2007; Pandey et al., 2008; Aryan et al., 2010, 2013; Geojith et al., 2011; Zhang et al., 2011; Yuan et al., 2014). LAMP relies upon an auto-cycling strand displacement DNA synthesis and is more tolerant to the presence of inhibitory substances such as blood, serum, plasma or heparin (Notomi et al., 2000). The reaction runs very rapidly in the presence of template nucleic acids and deoxynucleoside triphosphates, at a constant temperature (usually between 60 and 65°C), and provides high amplification efficiency with a detection limit and specificity comparable to those of standard PCR. The detection of amplified products by electrophoresis is not practical outside the laboratory but disposable generic strips for the lateral flow detection of nucleic acids, although still very expensive, are already commercially available (Chowdry et al., 2014). These chromatographic strips can detect biotin-labeled DNA fragments hybridized with complementary FITC-labeled probes. FITC is detected in the strips by the formation of complexes with gold-conjugated anti-FITC antibodies. Our group is exploring the integration of LAMP with these chromatographic strips for detecting veterinary-relevant MTC members, particularly *M. bovis*, by targeting specific RD elements, aiming the development of novel equipment-free molecular diagnostic kits for bovine tuberculosis (Costa et al., unpublished).

Other technological fields experiencing great developments are microfluidics and nanotechnology (Granberg et al., 2014; Teles and Fonseca, 2014). The procedures required for the molecular detection of pathogens in biological samples, including nucleic acids extraction and detection of specific genomic targets can all be conceptually integrated into miniaturized microfluidic systems, enabling the development of promising molecular diagnostics devices commonly referred to as Lab-on-a-Chip (Granberg et al., 2014; Teles and Fonseca, 2014). Particularly interesting have been the internationally significant research efforts invested in the development of a next generation of low-cost microfluidic paper-based analytical devices (Martinez et al., 2007, 2010; Vella et al., 2012; Rozand, 2014), also known as paperfluidics or Lab-on-Paper (Costa et al., 2013b). These devices are based on the definition of microchannels and test zones on hydrophilic paper via the patterning of walls of hydrophobic polymers, photoresist, or wax. Movement of fluids within channels is by capillarity and thus independent multi-analyte assays can be conducted that do not require use of pipettes, pumps, or electric energy. Multiple detection zones for different target compounds are created by deposition of reagents such as enzymes and antibodies on the paper surface (Martinez et al., 2010). When the sample reaches the detection zone, a reaction occurs and a color is developed. Assays for detecting total proteins, cholesterol, and glucose in fluids have been demonstrated and more specific biorecognition assays usually involve antibody-antigen interactions (Martinez et al., 2010; Vella et al., 2012; Costa et al., 2013b). We and other groups are trying to adapt paperfluidics platforms for the development of nucleic acid testing assays (Araújo et al., 2012; Veigas et al., 2012; Costa et al., 2013b), a major largely unsolved problem being the efficiency of immobilization processes of nucleic acid probes on the cellulosic paper matrices. In this context, very recently, Rosa and colleagues described a novel methodology to capture labeled-DNA strands and hybrids on paper, using MTC nucleic acid templates as model, via the anchoring of antibodies with a fusion protein that combines a carbohydrate binding module (CBM), with high affinity to cellulose, and the ZZ fragment of the staphyloccocal protein A, which recognizes IgG antibodies via their Fc portion (Rosa et al., 2014). Antibodies immobilized on paper matrices via CBM-ZZ were able to capture appropriately labeled (biotin, fluorescein) DNA strands and DNA hybrids. The efficiency of the capture of labeled-DNA by this strategy was significantly higher when compared with a physical adsorption method, which constitutes an important step toward the development of affordable paperfluidics-based molecular diagnostic tests.

Nanotechnologies have also sparked interest in the development of new diagnostic tests and biosensors, including nucleic acid testing strategies, taking advantage of the unique properties that many materials exhibit at the nanoscale (Teles and Fonseca, 2014). Gold-nanoparticles, in particular, are already used frequently in research for the molecular diagnostics of MTC bacteria (Liandris et al., 2009; Costa et al., 2010, 2013b; Veigas et al., 2012). In an example, gold nanoparticles conjugated with oligonucleotide probes (gold-nanoprobes) allow the colorimetric detection of complementary DNA targets (Baptista et al., 2008). In solution, monodisperse gold-nanoprobes appear red and exhibit a Surface Plasmon Resonance (SPR) band around 520 nm. In contrast, a solution containing aggregated gold-nanoprobes appears blue, corresponding to a characteristic shift in the SPR band to higher wavelengths. The presence of complementary DNA targets prevents aggregation of the gold-nanoprobes and the solution remains red; non-complementary targets do not prevent gold-nanoprobe aggregation resulting in a visible color change to blue. This gold-nanoprobe non-cross-linking method has been applied for the colorimetric identification of *M. bovis* and other MTC mycobacteria, for example based in the detection of their PCR-amplified *gyrB* genes (Costa et al., 2010).

Finally, over the last years, advanced high-throughput sequencing technologies, and associated bioinformatics, have generated massive amounts of sequence information, also for relevant viral, parasitic and bacterial animal pathogens (Cantacessi et al., 2014; Van Borm et al., 2014), with the possibility to generate highly redundant genome or metagenomic sequences for just a few tens or hundreds of dollars (Joseph and Read, 2010; Köser et al., 2012). In this context, there are tens or even hundreds of genome sequences currently available for an increasing number of individual microbial pathogenic species, with a particular focus for MTC members such as *M. tuberculosis* and *M. bovis* (Roetzer et al., 2013; Coll et al., 2014). The analysis of SNPs and other genetic polymorphisms information derived from Whole Genome Sequencing (WGS) studies is allowing to uncover the natural variation of MTC populations and the relation of these pathogens with their hosts, including virulence, drug susceptibility and immune modulator determinants relevant to the clinical manifestations of disease (Ford et al., 2012; Coll et al., 2014). With the rapid decrease in DNA sequencing costs, it is foreseen that WGS and metagenomics technologies will play an increasing role in clinical microbiology laboratories, particularly for molecular epidemiology studies (for surveillance and outbreak investigation) and genotypic antimicrobial susceptibility testing, with a focus for fastidious microorganisms such as MTC species (Biek et al., 2012; Köser et al., 2012; Roetzer et al., 2013). High throughput sequencing technologies and metagenomics may be also useful for detecting novel pathogens, or variants of known pathogens, that leads to false negative results using the standard diagnostic tests (Köser et al., 2012). In the coming years we will continue to experience tremendous developments in high-throughput sequencing technologies that will shape the design of novel diagnostics and disease intervention strategies, including in the veterinary field (Cantacessi et al., 2014; Van Borm et al., 2014).

## SWOT analysis for MTC nucleic acid testing in veterinary laboratories

Despite the great advantages normally associated with nucleic acid testing technologies, for example in terms of specificity, sensitivity and speed of response, veterinary laboratories face several challenges in the implementation and use of these methods (Cunha and Inácio, 2014). In Table 1 we disclose a self-explanatory SWOT analysis summarizing our view on the current most relevant Strengths, Weaknesses, Opportunities, and Threats associated to the use of nucleic acid testing strategies to uncover MTC members in veterinary diagnosis laboratories.

**Table 1:** **SWOT analysis for MTC nucleic acid testing in veterinary laboratories**.

**Strengths**	**Weaknesses**
Overall higher diagnostic specificities and sensitivities High discriminatory power Short time-to-result High sample throughput Multiplexing capability Possibility to target antimicrobial resistance and virulence associated genes or polymorphisms Possibility to quantify target nucleic acids in samples Wide range of public databases and bioinformatics tools available for analysis of molecular data	Requirement for more expensive facilities, reagents and consumables Requirement for more technically complex procedures Potential for the occurrence of false positives due to contamination by target nucleic acids Potential for the occurrence of false negatives due to difficulties in extracting and purifying MTC nucleic acids from tissues Nucleic acid testing does not usually differentiate between viable and nonviable mycobacteria
**Opportunities**	**Threats**
Growing demand for diagnostic tests for food-producing animals Demand for “point-of-care” diagnostic tests Educational efforts to disseminate nucleic acid testing Automation of nucleic acid tests Demand for more efficient DNA extraction and purification assays Microfluidics and nanotechnologies are becoming more mature for developing alternative nucleic acid tests Next generation sequencing and the “population genomics era” Increasing amounts of genetic information available from public databases Less strict regulations in the veterinary sector can facilitate a more rapid adoption of new diagnostic technologies	Pressure to keep a low price per analysis in the veterinary sector Lack of harmonization of nucleic acid testing between different laboratories Conservatism of medical personnel and technicians in the adoption and use of new diagnostics technologies Difficulty in finding good gold standard diagnostic methods to validate novel nucleic acid tests

## Conclusions

Nucleic acid testing will be increasingly used in the veterinary context. Identification of MTC cultures is relatively straightforward when using nucleic acid testing approaches but the molecular detection of these organisms directly in tissue specimens remains extremely challenging. A problem still largely unsolved concerns the processes of extraction and purification of mycobacterial nucleic acids from biological matrices. Noteworthy, the use of re-emerging nested PCR strategies may prove useful for detecting *M. bovis* and other MTC members in animal tissue specimens. Isothermal DNA amplification technologies, microfluidics and nanotechnology may also soon provide the basis for more efficient and widespread low-cost diagnostic devices targeting MTC organisms. Next generation sequencing and high-throughput metagenomics are forecasted to greatly shape our future understanding about microbial pathogens and will contribute for designing enhanced diagnostics and intervention strategies. Yet, we do not anticipate the complete replacement of culture-based diagnostics, which is also subject to improvements, by nucleic acid testing technologies. On the contrary, the real added value of molecular diagnostics will be in their complementarily use with conventional microbiological tests, allowing the collection and analysis of multivariate phenotypic and genotypic characteristics of pathogenic microorganisms.

## Author contributions

All authors contributed substantially to the conception and preparation of this review, revised it critically, approved the final version to be published, and agreed to be accountable for all aspects of the work.

### Conflict of interest statement

The authors declare that the research was conducted in the absence of any commercial or financial relationships that could be construed as a potential conflict of interest.
